# Crystal structure of di­fluorido­{2-[(4-hy­droxy­phen­yl)diazen­yl]-3,5-di­methyl­pyrrolido}boron

**DOI:** 10.1107/S2056989018006229

**Published:** 2018-04-27

**Authors:** Huixiao Feng, Zhenming Yin

**Affiliations:** a College of Chemistry, Tianjin Key Laboratory of Structure and Performance for Functional Molecules, Tianjin Normal University, Tianjin 300387, People’s Republic of China; b Key Laboratory of Inorganic–Organic Hybrid Functional Materials Chemistry, (Tianjin Normal University), Ministry of Education, Tianjin 300387, People’s Republic of China

**Keywords:** azo­pyrrole, borondifluoride complex, crystal structure, hydrogen bond

## Abstract

The asymmetric unit of the title azo­pyrrole-BF_2_ complex, C_12_H_12_BF_2_N_3_O, contains two independent mol­ecules, which are linked by an O—H⋯O hydrogen bond. The dimers are further assembled into a one-dimensional ladder-like structure through O—H⋯F hydrogen bonds and stabilized by π–π inter­actions. The ladders are further linked by C—H⋯π contacts.

## Abstract 2

The asymmetric unit contains two independent mol­ecules, which are linked by an O—H⋯O hydrogen bond. The dimers are further assembled into one-dimensional ladder like structure through O—H⋯F hydrogen bonds and stabilized by π–π inter­actions. The ladders are further linked by C—H⋯π contacts.

## Chemical context   

Recently, some unique pyrrole-BF_2_-based dyes have emerged as alternatives to 4,4-di­fluoro-4-bora-3a,4a-di­aza-*s*-indacene (BODIPY) dyes because of their easy synthesis, lower symmetry and longer wavelengt absorption. Li *et al.* (2009 ▸) have synthesized a series of azo­pyrroles and their di­fluoro­boron complexes, which possess promising absorption properties. The potentials of a few BF_2_–azo­pyrrole complexes as sensitizers for dye-sensitized solar cells (DSSCs) have been evaluated (Mikroyannidis, Royd *et al.*, 2010 ▸). In the me­antime, some BF_2_–azo­pyrrole complexes have been used for the fabrication of bulk heterojunction solar cells (Mikroyannidis, Kabanakis *et al.*, 2010 ▸). A 2-(di­methyl­amino­phenyl­azo)-5-ethyl-pyrrole boron difluoride complex has been used as an OFF–ON–OFF-type three-stage binary pH switch (Lee *et al.*, 2012 ▸). Previously, we have reported the crystal structures of some azo­pyrrole compounds (Yin *et al.*, 2008 ▸; Li *et al.*, 2011 ▸). In an extension of this research, we report herein on the crystal structure of di­fluorido­{2-[(4-hy­droxy­phen­yl)diazen­yl]-3,5-di­methyl­pyrrolido}boron.
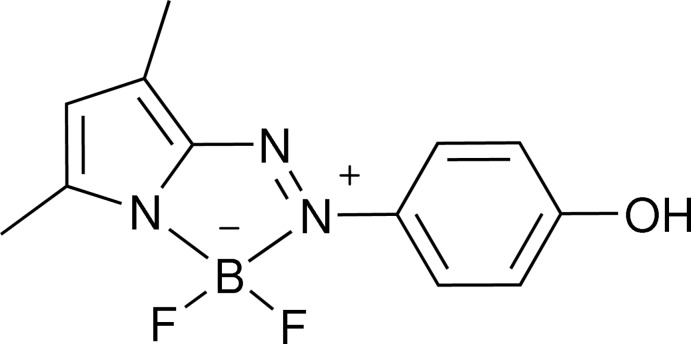


## Structural commentary   

**Figure 1:**
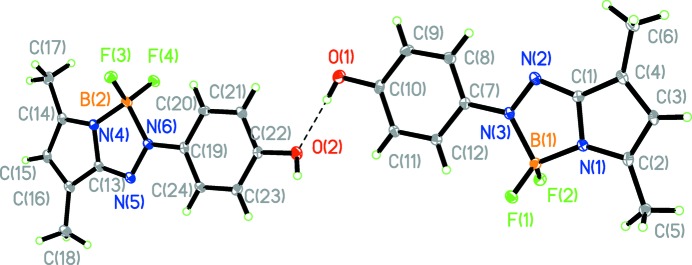
The asymmetric unit of the title compound, with displacement ellipsoids drawn at the 30% probability level. The O—H⋯O hydrogen bond is shown as a dashed line.

The asymmetric unit contains two independent mol­ecules, which show slight differences in some bond lengths [*e.g*. O1—C10 and O2—C22 = 1.358 (3) and 1.382 (3) Å, respectively; Table 1 ▸] and torsion angles [N2—N3—C7—C12 and N5—N6—C19—C20 are −171.1 (2) and 177.9 (2)°, respectively]. The r.m.s. deviation for fitting two molecules = 0.055 Å. The two mol­ecules are linked by the O1—H1⋯O2 hydrogen bond (Fig. 1 ▸, Table 2 ▸). The torsion angles between benzene rings and neighboring pyrrole rings in the N1- and N4-containing mol­ecules are 9.43 (12) and 1.34 (12)°, respectively. Each boron atom is four-coordinated by two fluorine atoms, a pyrrole N atom and an azo N atom. The B—N bond distances vary from 1.537 (3) to 1.618 (3) Å (Table 1 ▸). The B—N_pyrrole_ bonds are shorter than the B—N_azo_ bonds. The two N—N bonds each adopt a *trans* conformation and at 1.318 (3) and 1.312 (3) Å are much longer than that in the structure of the free azo­pyrrole ligand (Yin *et al.*, 2008 ▸). In addition, the C1—C4, C2—C3, C13—C16 and C14—C15 bonds are lengthened, while the C3—C4 and C15—C16 bonds are shortened compared to the normal bond lengths in pyrrole. This indicates that the azo­pyrrole moiety of the title compound must be in the hydrazone form (Chen *et al.*, 2014 ▸).

## Supra­molecular features and Hirshfeld analysis   

**Figure 2:**
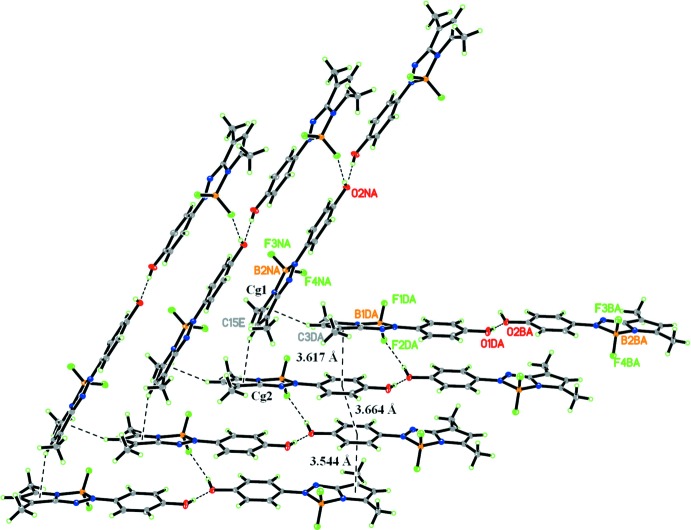
Part of the crystal packing showing mol­ecules linked by O—H⋯O and O—H⋯F hydrogen bonds, π–π inter­actions and C—H⋯π contacts.

The two conformers also show supra­molecular differences. One of the conformers only has a hydrogen bond between its hydroxyl group and that of the other conformer mol­ecule (Fig. 1 ▸), whereas the hydroxyl group in the other conformer is also involved in inter­molecular O—H⋯F inter­actions (Fig. 2 ▸, Table 2 ▸), forming a one-dimensional ladder-like structure along [100]. In the ladder structure, the mol­ecules are arranged in a parallel manner through π–π inter­actions [*Cg*1⋯*Cg*4(*x* − 1, *y*, *z*) = 3.544 (1) Å, *Cg*2⋯*Cg*3(1 + *x*, *y*, *z*) = 3.617 (1) Å and *Cg*3⋯*Cg*4(1 + *x*, *y*, *z*) = 3.664 (13) Å; *Cg*1, *Cg*2, *Cg*3 and *Cg*4 are the centroids of the N1/C1–C4, C7–C12 and C19–C24 rings, respectively]. The ladders assemble into a layer structure through C—H⋯π contacts (Table 2 ▸).

**Figure 3:**
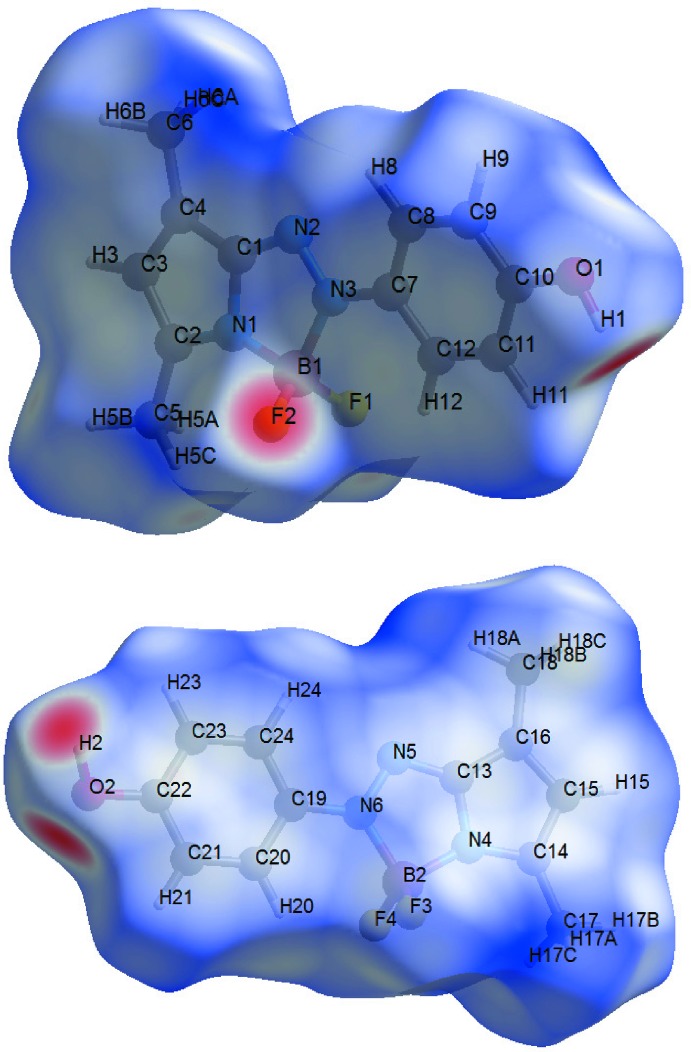
Hirshfeld surfaces of the two conformers mapped over *d*_norm_ in the range −0.614 to 1.350 a.u. The inter­molecular contacts can be seen in red regions.

The Hirshfeld surfaces of the two conformers were generated using *CrystalExplorer* (Turner *et al.*, 2017 ▸). Fig. 3 ▸ clearly shows that the two conformers are involved in different supra­molecular inter­actions.

## Database survey   

A search in the Cambridge Structural Database (Version 5.38; Groom *et al.*, 2016 ▸) for azo­pyrrole boron difluoride compounds returned two entries, 2,5-bis­(4-di­methyl­amino­phenyl­azo)pyrrole boron difluoride (Li *et al.*, 2009 ▸) and 2-(di­methyl­amino­phenyl­azo)-5-ethyl-pyrrole boron difluoride (Lee *et al.*, 2012 ▸). In both, the boron atoms have same coordination as in the title compound. The N—N bonds also adopt *trans* conformations and their lengths [1.322 (2) and 1.310 (1) Å] are comparable to those in the title compound.

## Synthesis and crystallization   

To a solution of 2-(4-hy­droxy­lphenyl­azo)-3,5-dimethyl-1-*H*-pyrrole (2 mmol, 0.43g) and tri­ethyl­amine (6 mL) in dry di­chloro­methane (15 mL) was slowly added boron trifluoride ethyl ether (2 mL). The resulting solution was stirred for 40 min, and then saturated potassium carbonate solution was added and stirred for 30 minutes. The resulting solution was extracted with ethyl acetate (10 mL × 3) and evaporated under vacuum to dryness. The residue was purified by column chromatography, eluting with ethyl acetate and petroleum ether (*v*/*v* = 1:14), to give a dark-green product, m.p. = 405 K. Yield 65%. ^1^H NMR (400 MHz, DMSO-*d*_6_): δ 10.118 (*s*, 1H, –OH), 7.548–7.526 (*d*, 2H, *J* = 8.8Hz, Ar–CH), 6.920–6.897(*d*, 2H, *J* = 9.2Hz, Ar–CH), 6.342 (*s*, 1H, pyrrole–CH), 2.371(*s*, 3H, –CH_3_), 2.314 (*s*, 3H, –CH_3_). Suitable crystals for X-ray diffraction analysis were obtained by the slow evaporation of an CHCl_3_/CH_3_OH solution of the title compound.

## Refinement   

Crystal data, data collection and structure refinement details are summarized in Table 3 ▸. OH H atoms were located from difference-Fourier maps and refined freely. Other H atoms were placed in calculated positions (C—H = 0.93 or 0.96 Å) and refined using a riding model, with *U*_iso_(H) = 1.2*U*_eq_(C) or 1.5*U*_eq_(C-meth­yl).

## Supplementary Material

Crystal structure: contains datablock(s) I. DOI: 10.1107/S2056989018006229/ex2007sup1.cif

Structure factors: contains datablock(s) I. DOI: 10.1107/S2056989018006229/ex2007Isup2.hkl

CCDC reference: 1839158

Additional supporting information:  crystallographic information; 3D view; checkCIF report

## Figures and Tables

**Table 1:** Selected bond lengths (Å)

F1—B1	1.369 (3)	F3—B2	1.368 (3)
F2—B1	1.401 (3)	F4—B2	1.380 (3)
O1—C10	1.358 (3)	O2—C22	1.382 (3)
N1—C1	1.377 (3)	N4—C13	1.380 (3)
N1—C2	1.356 (3)	N4—C14	1.353 (3)
N1—B1	1.537 (3)	N4—B2	1.545 (3)
N2—N3	1.318 (3)	N5—N6	1.312 (3)
N2—C1	1.343 (3)	N5—C13	1.338 (3)
N3—C7	1.406 (3)	N6—C19	1.416 (3)
N3—B1	1.613 (3)	N6—B2	1.618 (3)
C1—C4	1.411 (3)	C13—C16	1.415 (3)
C2—C3	1.405 (3)	C14—C15	1.408 (3)
C3—C4	1.389 (3)	C15—C16	1.389 (3)

**Table 2:** Hydrogen-bond geometry (Å, °) *Cg*2 and *Cg*6 are the centroids of the N4/C13–C16 and N1/C1–C4 rings, respectively.

*D*—H⋯*A*	*D*—H	H⋯*A*	*D*⋯*A*	*D*—H⋯*A*
O1—H1⋯O2	0.82	1.98	2.797 (2)	178
O2—H2⋯F2^i^	0.82	2.06	2.812 (2)	152
C3—H3⋯*Cg*1^ii^	0.93	2.62	3.501 (2)	158
C15—H15⋯*Cg*2^iii^	0.93	2.63	3.506 (2)	157

Symmetry codes: (i) 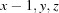; (ii) 
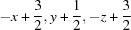
; (iii) 
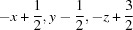
.

**Table 3:** Experimental details

Crystal data	
Chemical formula	C_12_H_12_BF_2_N_3_O
*M*_r_	263.06
Crystal system, space group	Monoclinic, *P*2_1_/*n*
Temperature (K)	100
*a*, *b*, *c* (Å)	6.8080 (4), 24.8217 (18), 14.4744 (9)
β (°)	100.489 (6)
*V* (Å^3^)	2405.1 (3)
*Z*	8
Radiation type	Mo *K*α
μ (mm^−1^)	0.12
Crystal size (mm)	0.25 × 0.22 × 0.2

Data collection	
Diffractometer	Rigaku Oxford Diffraction SuperNova, Dual, Cu at zero, Atlas S2
Absorption correction	Multi-scan (*CrysAlis PRO*; Rigaku OD, 2015 ▸)
*T*_min_, *T*_max_	0.680, 1.000
No. of measured, independent and observed [*I* > 2σ(*I*)] reflections	11903, 4228, 3277
*R*_int_	0.043
(sin θ/λ)_max_ (Å^−1^)	0.595

Refinement	
*R*[*F* ^2^ > 2σ(*F* ^2^)], *wR*(*F* ^2^), *S*	0.048, 0.116, 1.06
No. of reflections	4228
No. of parameters	349
H-atom treatment	H-atom parameters constrained
Δρ_max_, Δρ_min_ (e Å^−3^)	0.25, −0.26

Computer programs: *CrysAlis PRO* (Rigaku OD, 2015 ▸), *SHELXT* (Sheldrick, 2015*a* ▸), *SHELXL2014* (Sheldrick, 2015*b* ▸), *OLEX2* (Dolomanov *et al.*, 2009 ▸).

## supplementary crystallographic information

### Crystal data

**Table:**

C_12_H_12_BF_2_N_3_O	*F*(000) = 1088
*M**_r_* = 263.06	*D*_x_ = 1.453 Mg m^−^^3^
Monoclinic, *P*2_1_/*n*	Mo *K*α radiation, λ = 0.71073 Å
*a* = 6.8080 (4) Å	Cell parameters from 3432 reflections
*b* = 24.8217 (18) Å	θ = 4.1–28.6°
*c* = 14.4744 (9) Å	µ = 0.12 mm^−^^1^
β = 100.489 (6)°	*T* = 100 K
*V* = 2405.1 (3) Å^3^	Block, dark green
*Z* = 8	0.25 × 0.22 × 0.2 mm

### Data collection

**Table:**

Rigaku Oxford Diffraction SuperNova, Dual, Cu at zero, Atlas S2 diffractometer	4228 independent reflections
Radiation source: micro-focus sealed X-ray tube, SuperNova (Mo) X-ray Source	3277 reflections with *I* > 2σ(*I*)
Mirror monochromator	*R*_int_ = 0.043
Detector resolution: 5.2740 pixels mm^-1^	θ_max_ = 25.0°, θ_min_ = 3.2°
ω scans	*h* = −7→8
Absorption correction: multi-scan (CrysAlis PRO; Rigaku OD, 2015)	*k* = −29→29
*T*_min_ = 0.680, *T*_max_ = 1.000	*l* = −17→15
11903 measured reflections	

### Refinement

**Table:**

Refinement on *F*^2^	Primary atom site location: dual
Least-squares matrix: full	Hydrogen site location: inferred from neighbouring sites
*R*[*F*^2^ > 2σ(*F*^2^)] = 0.048	H-atom parameters constrained
*wR*(*F*^2^) = 0.116	*w* = 1/[σ^2^(*F*_o_^2^) + (0.0388*P*)^2^ + 1.6938*P*] where *P* = (*F*_o_^2^ + 2*F*_c_^2^)/3
*S* = 1.06	(Δ/σ)_max_ < 0.001
4228 reflections	Δρ_max_ = 0.25 e Å^−^^3^
349 parameters	Δρ_min_ = −0.26 e Å^−^^3^
0 restraints	

### Special details

**Table:**

Geometry. All esds (except the esd in the dihedral angle between two l.s. planes) are estimated using the full covariance matrix. The cell esds are taken into account individually in the estimation of esds in distances, angles and torsion angles; correlations between esds in cell parameters are only used when they are defined by crystal symmetry. An approximate (isotropic) treatment of cell esds is used for estimating esds involving l.s. planes.

### Fractional atomic coordinates and isotropic or equivalent isotropic displacement parameters (Å^2^)

**Table:**

	*x*	*y*	*z*	*U*_iso_*/*U*_eq_	
F1	1.31342 (18)	0.53429 (5)	0.89956 (9)	0.0210 (3)	
F2	1.46735 (18)	0.45513 (5)	0.87853 (8)	0.0205 (3)	
O1	0.5707 (2)	0.40021 (7)	0.61520 (11)	0.0272 (4)	
H1	0.5225	0.3893	0.6596	0.041*	
N1	1.5952 (3)	0.53806 (7)	0.81633 (12)	0.0156 (4)	
N2	1.3878 (3)	0.52607 (8)	0.67448 (13)	0.0178 (4)	
N3	1.2967 (3)	0.50217 (7)	0.73622 (13)	0.0162 (4)	
C1	1.5603 (3)	0.54666 (9)	0.72074 (15)	0.0162 (5)	
C2	1.7710 (3)	0.56211 (9)	0.85288 (16)	0.0166 (5)	
C3	1.8484 (3)	0.58566 (9)	0.77884 (16)	0.0182 (5)	
H3	1.9686	0.6043	0.7852	0.022*	
C4	1.7176 (3)	0.57669 (9)	0.69470 (16)	0.0180 (5)	
C5	1.8540 (3)	0.56219 (10)	0.95504 (15)	0.0211 (5)	
H5A	1.8228	0.5958	0.9820	0.032*	
H5B	1.9964	0.5578	0.9645	0.032*	
H5C	1.7965	0.5331	0.9848	0.032*	
C6	1.7354 (4)	0.59469 (11)	0.59812 (16)	0.0257 (6)	
H6A	1.6381	0.5762	0.5528	0.038*	
H6B	1.8669	0.5866	0.5869	0.038*	
H6C	1.7125	0.6328	0.5926	0.038*	
C7	1.1152 (3)	0.47520 (9)	0.70490 (15)	0.0163 (5)	
C8	1.0407 (3)	0.46718 (9)	0.60966 (16)	0.0198 (5)	
H8	1.1142	0.4786	0.5650	0.024*	
C9	0.8590 (3)	0.44247 (10)	0.58158 (16)	0.0223 (5)	
H9	0.8086	0.4380	0.5179	0.027*	
C10	0.7495 (3)	0.42404 (9)	0.64779 (16)	0.0190 (5)	
C11	0.8254 (3)	0.43105 (9)	0.74289 (16)	0.0179 (5)	
H11	0.7541	0.4185	0.7876	0.021*	
C12	1.0063 (3)	0.45662 (9)	0.77097 (15)	0.0170 (5)	
H12	1.0560	0.4615	0.8346	0.020*	
B1	1.4183 (4)	0.50677 (11)	0.84252 (18)	0.0174 (6)	
F3	−0.50768 (19)	0.27493 (6)	0.54470 (9)	0.0247 (3)	
F4	−0.33107 (18)	0.19699 (6)	0.55925 (9)	0.0237 (3)	
O2	0.3980 (2)	0.36293 (7)	0.76431 (11)	0.0226 (4)	
H2	0.4090	0.3820	0.8112	0.034*	
N4	−0.6118 (3)	0.20404 (8)	0.64279 (13)	0.0172 (4)	
N5	−0.4053 (3)	0.23598 (8)	0.77401 (13)	0.0176 (4)	
N6	−0.3216 (3)	0.25089 (8)	0.70328 (12)	0.0167 (4)	
C13	−0.5729 (3)	0.20907 (9)	0.73934 (15)	0.0168 (5)	
C14	−0.7850 (3)	0.17649 (9)	0.61832 (16)	0.0188 (5)	
C15	−0.8566 (3)	0.16381 (9)	0.70106 (16)	0.0198 (5)	
H15	−0.9737	0.1451	0.7036	0.024*	
C16	−0.7251 (3)	0.18365 (9)	0.77825 (16)	0.0193 (5)	
C17	−0.8717 (3)	0.16370 (11)	0.51897 (17)	0.0261 (6)	
H17A	−0.9208	0.1961	0.4867	0.039*	
H17B	−0.9797	0.1386	0.5171	0.039*	
H17C	−0.7707	0.1481	0.4889	0.039*	
C18	−0.7344 (4)	0.17973 (10)	0.88049 (16)	0.0254 (6)	
H18A	−0.6142	0.1943	0.9169	0.038*	
H18B	−0.7476	0.1426	0.8971	0.038*	
H18C	−0.8474	0.1997	0.8931	0.038*	
C19	−0.1401 (3)	0.28017 (9)	0.72225 (15)	0.0155 (5)	
C20	−0.0500 (3)	0.29409 (9)	0.64649 (16)	0.0186 (5)	
H20	−0.1105	0.2848	0.5857	0.022*	
C21	0.1291 (3)	0.32173 (9)	0.66166 (16)	0.0182 (5)	
H21	0.1898	0.3308	0.6112	0.022*	
C22	0.2179 (3)	0.33579 (9)	0.75205 (16)	0.0170 (5)	
C23	0.1294 (3)	0.32146 (9)	0.82758 (16)	0.0183 (5)	
H23	0.1907	0.3306	0.8883	0.022*	
C24	−0.0496 (3)	0.29372 (9)	0.81286 (15)	0.0180 (5)	
H24	−0.1090	0.2842	0.8635	0.022*	
B2	−0.4440 (4)	0.23212 (11)	0.60191 (18)	0.0192 (6)	

### Atomic displacement parameters (Å^2^)

**Table:**

	*U*^11^	*U*^22^	*U*^33^	*U*^12^	*U*^13^	*U*^23^
F1	0.0209 (7)	0.0223 (8)	0.0214 (7)	−0.0015 (6)	0.0079 (5)	−0.0030 (6)
F2	0.0227 (7)	0.0171 (7)	0.0211 (7)	−0.0029 (6)	0.0027 (5)	0.0025 (6)
O1	0.0236 (9)	0.0309 (11)	0.0260 (9)	−0.0135 (8)	0.0014 (7)	0.0018 (8)
N1	0.0156 (9)	0.0132 (10)	0.0185 (10)	0.0009 (8)	0.0048 (7)	−0.0001 (8)
N2	0.0182 (10)	0.0144 (10)	0.0221 (10)	−0.0002 (8)	0.0070 (8)	0.0008 (8)
N3	0.0148 (9)	0.0148 (10)	0.0195 (10)	−0.0007 (8)	0.0047 (8)	0.0007 (8)
C1	0.0174 (11)	0.0136 (12)	0.0176 (12)	0.0017 (10)	0.0031 (9)	0.0001 (10)
C2	0.0141 (11)	0.0117 (12)	0.0243 (12)	0.0026 (9)	0.0040 (9)	−0.0012 (10)
C3	0.0148 (11)	0.0128 (12)	0.0277 (13)	−0.0022 (9)	0.0058 (9)	−0.0003 (10)
C4	0.0182 (12)	0.0129 (12)	0.0248 (12)	0.0002 (10)	0.0087 (9)	0.0005 (10)
C5	0.0204 (12)	0.0209 (13)	0.0218 (12)	−0.0014 (10)	0.0037 (9)	0.0011 (10)
C6	0.0244 (13)	0.0275 (15)	0.0262 (13)	−0.0037 (11)	0.0073 (10)	0.0049 (11)
C7	0.0163 (11)	0.0117 (12)	0.0207 (12)	−0.0006 (9)	0.0033 (9)	0.0013 (10)
C8	0.0220 (12)	0.0185 (13)	0.0198 (12)	−0.0030 (10)	0.0061 (9)	0.0021 (10)
C9	0.0265 (13)	0.0220 (14)	0.0169 (12)	−0.0048 (11)	0.0002 (9)	−0.0001 (10)
C10	0.0146 (11)	0.0152 (13)	0.0263 (13)	−0.0016 (10)	0.0009 (9)	0.0007 (10)
C11	0.0161 (11)	0.0167 (12)	0.0219 (12)	0.0019 (10)	0.0066 (9)	0.0032 (10)
C12	0.0187 (12)	0.0155 (12)	0.0168 (11)	−0.0004 (10)	0.0032 (9)	−0.0006 (10)
B1	0.0169 (13)	0.0168 (14)	0.0185 (13)	−0.0009 (11)	0.0031 (10)	0.0016 (11)
F3	0.0265 (7)	0.0242 (8)	0.0230 (7)	−0.0026 (6)	0.0035 (6)	0.0065 (6)
F4	0.0227 (7)	0.0267 (8)	0.0234 (7)	−0.0033 (6)	0.0089 (6)	−0.0067 (6)
O2	0.0197 (8)	0.0208 (10)	0.0278 (9)	−0.0056 (7)	0.0056 (7)	−0.0043 (8)
N4	0.0170 (10)	0.0160 (11)	0.0190 (10)	−0.0010 (8)	0.0049 (8)	0.0002 (8)
N5	0.0191 (10)	0.0141 (10)	0.0211 (10)	0.0006 (8)	0.0073 (8)	−0.0002 (8)
N6	0.0181 (10)	0.0139 (10)	0.0193 (10)	−0.0016 (8)	0.0064 (8)	0.0009 (8)
C13	0.0171 (12)	0.0132 (12)	0.0210 (12)	0.0021 (10)	0.0063 (9)	0.0006 (10)
C14	0.0159 (11)	0.0142 (12)	0.0266 (13)	0.0009 (10)	0.0047 (9)	0.0010 (10)
C15	0.0143 (11)	0.0153 (12)	0.0309 (13)	−0.0009 (10)	0.0065 (10)	0.0020 (11)
C16	0.0208 (12)	0.0137 (12)	0.0250 (12)	0.0029 (10)	0.0084 (10)	0.0030 (10)
C17	0.0200 (12)	0.0292 (15)	0.0280 (14)	−0.0039 (11)	0.0018 (10)	0.0011 (12)
C18	0.0283 (13)	0.0231 (14)	0.0275 (13)	0.0005 (11)	0.0123 (10)	0.0035 (11)
C19	0.0148 (11)	0.0101 (12)	0.0220 (12)	−0.0003 (9)	0.0046 (9)	0.0000 (10)
C20	0.0225 (12)	0.0171 (13)	0.0162 (11)	−0.0015 (10)	0.0039 (9)	−0.0007 (10)
C21	0.0197 (12)	0.0147 (12)	0.0223 (12)	0.0007 (10)	0.0094 (9)	0.0025 (10)
C22	0.0145 (11)	0.0130 (12)	0.0237 (12)	0.0001 (9)	0.0039 (9)	−0.0003 (10)
C23	0.0196 (12)	0.0146 (12)	0.0191 (12)	0.0001 (10)	−0.0004 (9)	−0.0028 (10)
C24	0.0194 (12)	0.0169 (13)	0.0193 (12)	0.0005 (10)	0.0079 (9)	0.0018 (10)
B2	0.0203 (13)	0.0203 (15)	0.0178 (13)	−0.0031 (12)	0.0059 (10)	0.0004 (12)

### Geometric parameters (Å, º)

**Table:**

F1—B1	1.369 (3)	F3—B2	1.368 (3)
F2—B1	1.401 (3)	F4—B2	1.380 (3)
O1—H1	0.8200	O2—H2	0.8200
O1—C10	1.358 (3)	O2—C22	1.382 (3)
N1—C1	1.377 (3)	N4—C13	1.380 (3)
N1—C2	1.356 (3)	N4—C14	1.353 (3)
N1—B1	1.537 (3)	N4—B2	1.545 (3)
N2—N3	1.318 (3)	N5—N6	1.312 (3)
N2—C1	1.343 (3)	N5—C13	1.338 (3)
N3—C7	1.406 (3)	N6—C19	1.416 (3)
N3—B1	1.613 (3)	N6—B2	1.618 (3)
C1—C4	1.411 (3)	C13—C16	1.415 (3)
C2—C3	1.405 (3)	C14—C15	1.408 (3)
C2—C5	1.484 (3)	C14—C17	1.486 (3)
C3—H3	0.9300	C15—H15	0.9300
C3—C4	1.389 (3)	C15—C16	1.389 (3)
C4—C6	1.493 (3)	C16—C18	1.496 (3)
C5—H5A	0.9600	C17—H17A	0.9600
C5—H5B	0.9600	C17—H17B	0.9600
C5—H5C	0.9600	C17—H17C	0.9600
C6—H6A	0.9600	C18—H18A	0.9600
C6—H6B	0.9600	C18—H18B	0.9600
C6—H6C	0.9600	C18—H18C	0.9600
C7—C8	1.394 (3)	C19—C20	1.394 (3)
C7—C12	1.391 (3)	C19—C24	1.385 (3)
C8—H8	0.9300	C20—H20	0.9300
C8—C9	1.375 (3)	C20—C21	1.382 (3)
C9—H9	0.9300	C21—H21	0.9300
C9—C10	1.394 (3)	C21—C22	1.382 (3)
C10—C11	1.390 (3)	C22—C23	1.387 (3)
C11—H11	0.9300	C23—H23	0.9300
C11—C12	1.380 (3)	C23—C24	1.382 (3)
C12—H12	0.9300	C24—H24	0.9300
			
C10—O1—H1	109.5	C22—O2—H2	109.5
C1—N1—B1	109.05 (17)	C13—N4—B2	109.16 (18)
C2—N1—C1	107.60 (18)	C14—N4—C13	107.95 (18)
C2—N1—B1	143.30 (19)	C14—N4—B2	142.88 (19)
N3—N2—C1	108.06 (18)	N6—N5—C13	108.05 (18)
N2—N3—C7	119.34 (18)	N5—N6—C19	118.74 (18)
N2—N3—B1	113.07 (17)	N5—N6—B2	113.64 (17)
C7—N3—B1	127.56 (18)	C19—N6—B2	127.62 (18)
N1—C1—C4	110.47 (18)	N4—C13—C16	110.09 (19)
N2—C1—N1	114.66 (19)	N5—C13—N4	114.72 (19)
N2—C1—C4	134.9 (2)	N5—C13—C16	135.2 (2)
N1—C2—C3	108.23 (19)	N4—C14—C15	108.08 (19)
N1—C2—C5	122.6 (2)	N4—C14—C17	122.4 (2)
C3—C2—C5	129.2 (2)	C15—C14—C17	129.6 (2)
C2—C3—H3	125.3	C14—C15—H15	125.3
C4—C3—C2	109.40 (19)	C16—C15—C14	109.4 (2)
C4—C3—H3	125.3	C16—C15—H15	125.3
C1—C4—C6	127.1 (2)	C13—C16—C18	126.1 (2)
C3—C4—C1	104.30 (19)	C15—C16—C13	104.4 (2)
C3—C4—C6	128.6 (2)	C15—C16—C18	129.5 (2)
C2—C5—H5A	109.5	C14—C17—H17A	109.5
C2—C5—H5B	109.5	C14—C17—H17B	109.5
C2—C5—H5C	109.5	C14—C17—H17C	109.5
H5A—C5—H5B	109.5	H17A—C17—H17B	109.5
H5A—C5—H5C	109.5	H17A—C17—H17C	109.5
H5B—C5—H5C	109.5	H17B—C17—H17C	109.5
C4—C6—H6A	109.5	C16—C18—H18A	109.5
C4—C6—H6B	109.5	C16—C18—H18B	109.5
C4—C6—H6C	109.5	C16—C18—H18C	109.5
H6A—C6—H6B	109.5	H18A—C18—H18B	109.5
H6A—C6—H6C	109.5	H18A—C18—H18C	109.5
H6B—C6—H6C	109.5	H18B—C18—H18C	109.5
C8—C7—N3	121.8 (2)	C20—C19—N6	117.90 (19)
C12—C7—N3	118.89 (19)	C24—C19—N6	122.0 (2)
C12—C7—C8	119.3 (2)	C24—C19—C20	120.1 (2)
C7—C8—H8	119.9	C19—C20—H20	120.0
C9—C8—C7	120.1 (2)	C21—C20—C19	120.0 (2)
C9—C8—H8	119.9	C21—C20—H20	120.0
C8—C9—H9	119.7	C20—C21—H21	120.1
C8—C9—C10	120.6 (2)	C20—C21—C22	119.8 (2)
C10—C9—H9	119.7	C22—C21—H21	120.1
O1—C10—C9	117.5 (2)	O2—C22—C21	118.0 (2)
O1—C10—C11	123.1 (2)	O2—C22—C23	121.8 (2)
C11—C10—C9	119.4 (2)	C21—C22—C23	120.2 (2)
C10—C11—H11	120.0	C22—C23—H23	119.9
C12—C11—C10	120.0 (2)	C24—C23—C22	120.2 (2)
C12—C11—H11	120.0	C24—C23—H23	119.9
C7—C12—H12	119.7	C19—C24—H24	120.2
C11—C12—C7	120.6 (2)	C23—C24—C19	119.7 (2)
C11—C12—H12	119.7	C23—C24—H24	120.2
F1—B1—F2	110.33 (19)	F3—B2—F4	111.15 (19)
F1—B1—N1	114.5 (2)	F3—B2—N4	114.04 (19)
F1—B1—N3	112.16 (18)	F3—B2—N6	112.3 (2)
F2—B1—N1	114.19 (18)	F4—B2—N4	113.4 (2)
F2—B1—N3	109.65 (19)	F4—B2—N6	110.52 (18)
N1—B1—N3	95.14 (17)	N4—B2—N6	94.42 (16)
			
O1—C10—C11—C12	−178.6 (2)	O2—C22—C23—C24	−179.2 (2)
N1—C1—C4—C3	0.1 (3)	N4—C13—C16—C15	−0.5 (3)
N1—C1—C4—C6	−179.5 (2)	N4—C13—C16—C18	179.0 (2)
N1—C2—C3—C4	0.7 (3)	N4—C14—C15—C16	−0.2 (3)
N2—N3—C7—C8	8.1 (3)	N5—N6—C19—C20	177.9 (2)
N2—N3—C7—C12	−171.1 (2)	N5—N6—C19—C24	−0.4 (3)
N2—N3—B1—F1	117.8 (2)	N5—N6—B2—F3	118.5 (2)
N2—N3—B1—F2	−119.2 (2)	N5—N6—B2—F4	−116.8 (2)
N2—N3—B1—N1	−1.2 (2)	N5—N6—B2—N4	0.2 (2)
N2—C1—C4—C3	178.7 (3)	N5—C13—C16—C15	179.5 (2)
N2—C1—C4—C6	−0.8 (4)	N5—C13—C16—C18	−1.0 (4)
N3—N2—C1—N1	−0.3 (3)	N6—N5—C13—N4	0.2 (3)
N3—N2—C1—C4	−178.9 (2)	N6—N5—C13—C16	−179.8 (3)
N3—C7—C8—C9	−177.5 (2)	N6—C19—C20—C21	−178.7 (2)
N3—C7—C12—C11	178.5 (2)	N6—C19—C24—C23	178.8 (2)
C1—N1—C2—C3	−0.6 (2)	C13—N4—C14—C15	−0.1 (3)
C1—N1—C2—C5	178.5 (2)	C13—N4—C14—C17	−179.9 (2)
C1—N1—B1—F1	−116.2 (2)	C13—N4—B2—F3	−116.9 (2)
C1—N1—B1—F2	115.2 (2)	C13—N4—B2—F4	114.5 (2)
C1—N1—B1—N3	1.0 (2)	C13—N4—B2—N6	−0.1 (2)
C1—N2—N3—C7	−177.34 (19)	C13—N5—N6—C19	−179.84 (19)
C1—N2—N3—B1	1.0 (2)	C13—N5—N6—B2	−0.3 (2)
C2—N1—C1—N2	−178.58 (19)	C14—N4—C13—N5	−179.60 (19)
C2—N1—C1—C4	0.4 (3)	C14—N4—C13—C16	0.4 (3)
C2—N1—B1—F1	60.6 (4)	C14—N4—B2—F3	62.3 (4)
C2—N1—B1—F2	−68.0 (4)	C14—N4—B2—F4	−66.2 (4)
C2—N1—B1—N3	177.8 (3)	C14—N4—B2—N6	179.2 (3)
C2—C3—C4—C1	−0.4 (3)	C14—C15—C16—C13	0.5 (3)
C2—C3—C4—C6	179.1 (2)	C14—C15—C16—C18	−179.0 (2)
C5—C2—C3—C4	−178.4 (2)	C17—C14—C15—C16	179.6 (2)
C7—N3—B1—F1	−64.0 (3)	C19—N6—B2—F3	−62.0 (3)
C7—N3—B1—F2	59.0 (3)	C19—N6—B2—F4	62.7 (3)
C7—N3—B1—N1	176.9 (2)	C19—N6—B2—N4	179.7 (2)
C7—C8—C9—C10	−1.6 (4)	C19—C20—C21—C22	−0.4 (3)
C8—C7—C12—C11	−0.7 (3)	C20—C19—C24—C23	0.5 (3)
C8—C9—C10—O1	179.7 (2)	C20—C21—C22—O2	179.4 (2)
C8—C9—C10—C11	0.3 (4)	C20—C21—C22—C23	1.1 (3)
C9—C10—C11—C12	0.8 (3)	C21—C22—C23—C24	−1.0 (3)
C10—C11—C12—C7	−0.5 (3)	C22—C23—C24—C19	0.1 (3)
C12—C7—C8—C9	1.8 (4)	C24—C19—C20—C21	−0.4 (3)
B1—N1—C1—N2	−0.6 (3)	B2—N4—C13—N5	−0.1 (3)
B1—N1—C1—C4	178.34 (19)	B2—N4—C13—C16	179.92 (19)
B1—N1—C2—C3	−177.4 (3)	B2—N4—C14—C15	−179.3 (3)
B1—N1—C2—C5	1.7 (4)	B2—N4—C14—C17	0.9 (5)
B1—N3—C7—C8	−170.0 (2)	B2—N6—C19—C20	−1.6 (3)
B1—N3—C7—C12	10.8 (3)	B2—N6—C19—C24	−179.8 (2)

### Hydrogen-bond geometry (Å, º)

Cg2 and Cg6 are the centroids of the N4/C13–C16 and N1/C1–C4 rings, respectively.

**Table:**

*D*—H···*A*	*D*—H	H···*A*	*D*···*A*	*D*—H···*A*
O1—H1···O2	0.82	1.98	2.797 (2)	178
O2—H2···F2^i^	0.82	2.06	2.812 (2)	152
C3—H3···*Cg*1^ii^	0.93	2.62	3.501 (2)	158
C15—H15···*Cg*2^iii^	0.93	2.63	3.506 (2)	157

Symmetry codes: (i) *x*−1, *y*, *z*; (ii) −*x*+3/2, *y*+1/2, −*z*+3/2; (iii) −*x*+1/2, *y*−1/2, −*z*+3/2.
